# ACO/ARO/AIO-21 - Capecitabine-based chemoradiotherapy in combination with the IL-1 receptor antagonist anakinra for rectal cancer Patients: A phase I trial of the German rectal cancer study group

**DOI:** 10.1016/j.ctro.2022.04.003

**Published:** 2022-04-06

**Authors:** Maximilian Fleischmann, Markus Diefenhardt, Adele M. Nicolas, Franz Rödel, Michael Ghadimi, Ralf-Dieter Hofheinz, Florian R. Greten, Claus Rödel, Emmanouil Fokas, on behalf of the German Rectal Cancer Study Group

**Affiliations:** a Department of Radiation Oncology, University Hospital Johann Wolfgang Goethe University, 60590 Frankfurt, Germany; b Institute for Tumor Biology and Experimental Therapy, Georg-Speyer-Haus (GSH), 60596 Frankfurt/Main, Germany; c Frankfurt Cancer Institute, 60590 Frankfurt, Germany; d German Cancer Research Center (DKFZ), 69120 Heidelberg, Germany; e German Cancer Consortium (DKTK), Partner Site Frankfurt am Main, 60590 Frankfurt, Germany; f Department of General and Visceral Surgery, University Medical Center Göttingen, 37075 Göttingen, Germany; g Department of Medical Oncology, University Hospital Mannheim, 68167 Mannheim, Germany

**Keywords:** Rectal cancer, Anakinra, phase I, Chemoradiotherapy, Fibroblast, Interleukin-1, LARC, locally advanced rectal cancer, c/pCR, clinical/pathological complete response, DFS, disease-free survival, IL-1, interleukin-1, (i)CAFs, (inflammatory) cancer-associated fibroblasts, ECM, extracellular matrix, RA, receptor antagonist, CRT, chemoradiotherapy, MTD, maximum tolerated dose, Gy, Gray, DRE, digital rectal examination, MRI, magnetic resonance imaging, NOM, non-operative management, TME, total mesorectal excision, TNT, total-neoadjuvant therapy, CMS, consensus classification of molecular subtypes, PDO, patient-derived organoids, mrCRM, MRI-assessed circumferential resection margin, EMVI, extramural vascular invasion, APR, abdominoperineal resection, CT, computed tomography, DLT, dose-limiting toxicity, bid, *bis in die* (twice a day), W&W, watch & wait, IMRT, intensity modulated radiotherapy, VMAT, volumetric modulated arc therapy, GTV, gross tumor volume, ICH, International Council for Harmonisation of Technical Requirements for Registration of Pharmaceuticals for Human Use, CEA, carcinoembryonic antigen, TRG, tumor regression grading, CAPS, cryopyrin-associated periodic syndrome, VEGF-A, vascular endothelial growth factor A

## Abstract

**Purpose:**

Recent advances in the treatment algorithm of locally advanced rectal cancer (LARC) have significantly improved complete response (CR) rates and disease-free survival (DFS), but therapy resistance, with its substantial impact on outcomes and survival, remains a major challenge. Our group has recently unraveled a critical role of interleukin-1α (IL-1α) signaling in activating inflammatory cancer-associated fibroblasts (iCAFs) and mediating radiation-induced senescence, extracellular matrix (ECM) accumulation, and ultimately therapy resistance. We here summarize the recently initiated ACO/ARO/AIO-21 phase I trial, testing the IL-1 receptor antagonist (IL-1 RA) anakinra in combination with fluoropyrimidine-based chemoradiotherapy (CRT) for advanced rectal cancer.

**Methods/Design:**

The ACO/ARO/AIO-21 is an investigator-driven, prospective, open-labeled phase I drug-repurposing trial assessing the maximum tolerated dose (MTD) of capecitabine administered concurrently to standard preoperative radiotherapy (45 Gy in 25 fractions followed by 9 Gy boost in 5 fractions) in combination with fixed doses of the IL1-RA anakinra (100 mg, days −10 to 30). Capecitabine will be administered using a 3 + 3 dose-escalation design (500 mg/m^2^ bid; 650 mg/m^2^ bid; 825 mg/m^2^ bid, respectively) from day 1 to day 30. Response assessment including digital rectal examination (DRE), endoscopy and pelvic magnetic resonance imaging (MRI) is scheduled 10 weeks after completion of CRT. For patients achieving clinical complete response (cCR), primary non-operative management is provided. In case of non-cCR immediate total mesorectal excision (TME) will be performed. Primary endpoint of this phase I trial is the MTD of capecitabine.

**Discussion:**

Based on extensive preclinical research, the ACO/ARO/AIO-21 phase I trial will assess whether the IL-1RA anakinra can be safely combined with fluoropyrimidine-based CRT in rectal cancer. It will further explore the potential of IL-1 inhibition to overcome therapy resistance and improve response rates. A comprehensive translational research program will expand our understanding from a clinical perspective and may help translate the results into a randomized phase II trial.

## Highlights

• Response to chemoradiotherapy in rectal cancer is highly heterogeneous, ranging from complete response to tumor progression.
• Interleukin-1 signaling polarizes cancer-associated fibroblasts (CAF) towards an inflammatory phenotype and predisposes iCAFs to irradiation-induced senescence.
• Targeting interleukin-1 could potentially reconstitute the tumor microenvironment and improve therapy response.
• The ACO/ARO-AIO-21 phase I trial is testing the interleukin-1 receptor antagonist anakinra in combination with fluoropyrimidine-based chemoradiotherapy in rectal cancer.

## Background

Fluoropyrimidine-based preoperative chemoradiotherapy (CRT) and total mesorectal excision (TME) 6–10 weeks thereafter, followed by optional adjuvant chemotherapy, has been the standard treatment for patients with stage II and III UICC rectal cancer. In this setting, pathologic complete response (pCR) rates are in the range of 10%, 3-year local failure rates are in the range of 5%, distant recurrences occur in 25–30% of patients, while 3-year disease-free survival (DFS) is approximately 70% [1], [2], [3].

Recent developments in intensity, sequence, and timing of the treatment algorithm have led to a more personalized and multidisciplinary approach, resulting in total neoadjuvant therapy (TNT) concepts. Increasing pathological complete response (pCR) rates have resulted in a substantial paradigm shift with optional non-operative management (NOM) and organ preservation for patients with clinical complete response (cCR) [4], [5], [6].

However, response to CRT and, more recently, TNT is extremely heterogeneous and varies between cCR and tumor progression, with substantial impact on outcome and survival [7], [8]. In this context, the molecular mechanisms associated with therapy response or resistance in rectal cancer are poorly understood. The consensus classification of molecular subtypes (CMS) for colorectal cancer allows a more accurate categorization of molecular subtypes based on transcriptomic profiles, which in turn improves the prediction of therapy response and prognosis. CMS4 subtype tumors are characterized by a mesenchymal signature and are associated with an impaired survival, outlining the importance of the tumor microenvironment (TME) [9]. One of the most prominent and heterogeneous cell populations within the TME are cancer-associated fibroblasts (CAFs), influencing cancer cell survival and proliferation, extracellular matrix (EMC) remodeling, angiogenesis, metastatic spread and therapy resistance [10].

**Fig. 1:**
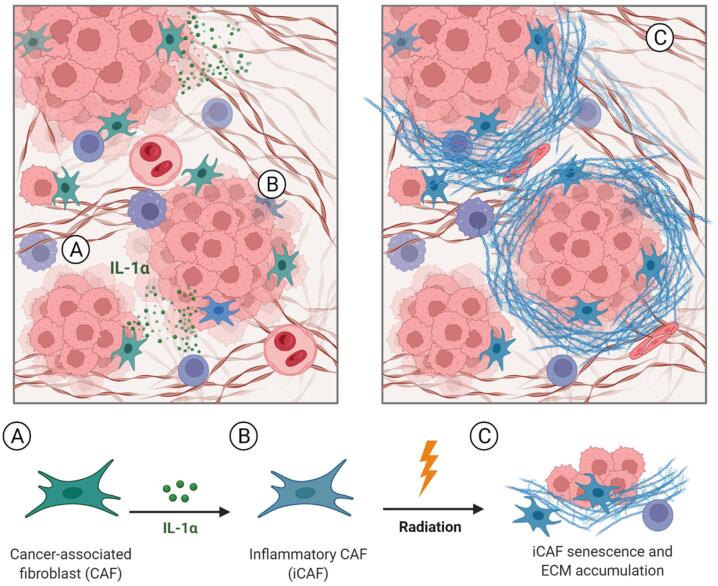
**Interleukin-1α signaling triggers inflammatory cancer-associated fibroblasts (iCAFs) which mediate therapy resistance via radiation-induced senescence and extracellular matrix (ECM) accumulation.** Proteomic analysis of pre-treatment biopsies from 61 rectal cancer patients have failed to reveal a protein expression profile predicting for pCR or non-pCR, indicating components of the TME could determine therapy response. Consequently, a strong enrichment of IL1/TNFα-dependent iCAFs was detected in patients with non-pCR (A). In addition, immunohistochemical analysis showed a high expression of decorin, a small cellular or pericellular matrix proteoglycan secreted by fibroblasts, which was associated with significantly worse DFS. To confirm the role of iCAFs in rectal cancer therapy resistance, a orthotopic mouse model or patients-derived organoids (PDO) was employed. First, therapy-resistant tumors were shown to induce inflammatory CAF polarization by intrinsic IL-1α signaling (B), demonstrating reciprocal crosstalk between tumor cells and CAFs. Second, IL-1α triggers nitrite-mediated oxidative DNA damage, thereby predisposing iCAFs to p53-mediated radiation-induced senescence. Finally, iCAF senescence led to ECM accumulation (C) and therapy resistance. Inhibition of IL-1 was shown to overcome CAF polarization and radiation-induced senescence, and thus resensitizing tumors to radiotherapy in a mouse model, while lower IL-1 receptor antagonist (IL-1RA) serum levels associated with a tumor-independent single nucleotide polymorphism (SNP) in rectal cancer patients were correlated to poor prognosis (not pictured). These results highlight the impact of a pro-inflammatory and tumor-promoting TME on therapy resistance and outcome in rectal cancer and provide a potential target for stroma repolarization and prevention of CAF senescence.

Our group has recently unraveled the critical role of inflammatory cancer-associated fibroblasts (iCAFs) and interleukin 1α (IL1α) signaling in therapy resistance, employing a murine rectal cancer model and patient-derived tumor organoids (PDO) [11]. IL-1 inhibition with the IL-1 receptor antagonist (IL-1RA) anakinra, a drug already approved for the treatment of patients with rheumatoid arthritis, led to CAF repolarization and significantly sensitized tumors to RT (Fig. 1). Thus, blockade of IL-1 signaling by anakinra may represent an attractive option to significantly improve response rates, organ preservation, and survival in rectal cancer. Based on these findings, the ACO/ARO/AIO-21 phase I trial has been recently initiated to investigate the safety and tolerability of standard fluoropyrimidine-based CRT in combination with the IL-1RA anakinra in rectal cancer. This drug-repurposing trial constitutes a prime example of translational research from bench to bedside..

## Methods/Design

### Setting

The ACO/ARO/AIO-21 phase I trial is an investigator-driven, prospective and open-labeled drug re-purposing trial assessing the safety and efficacy of the IL-1RA anakinra (Kineret ®) in combination with standard fluoropyrimidine-based CRT in patients with locally advanced rectal cancer. Female and male patients with histologically confirmed adenocarcinoma localized 0 – 12 cm from the anocutaneous line (measured by rigid rectoscopy) are eligible after local and distant staging procedures and after signing informed consent. For local staging, high-resolution magnetic resonance imaging (MRI) of the pelvis is mandatory. Patients with MRI-defined intermediate/high risk rectal cancer (≥cT3c/d, cT3 cN1, cT_any_ cN2, mrCRM+ (≤1 mm) or EMVI+), but not eligible for TNT, can be included. In addition, patients with MRI-defined low-risk rectal cancer (cT2-3a/b N0) ≤ 6 cm from the anocutaneous line that would require an abdominoperineal resection (APR) and/or permanent colostomy can also be included. Distant staging is performed with computed tomography (CT) scans of chest and abdomen. Table 1 summarizes the inclusion and exclusion criteria.

**Table 1:** Inclusion and exclusion criteria of the ACO/ARO/AIO-21 trial.

Inclusion Criteria	• Male and female patients with histologically confirmed diagnosis of rectal adenocarcinoma localized 0 – 12 cm from the anocutaneous line as measured by rigid rectoscopy (i.e. lower and middle third of the rectum) Staging requirements: High-resolution, thin-sliced (i.e. 3 mm) magnetic resonance imaging (MRI) of the pelvis is the mandatory local staging procedure. Patients with MRI-defined low risk rectal cancer with the presence of at least one of the following conditions: - cT2N0 or cT3a/bN0 tumors ≤ 6 cm from the anocutaneous line that would require abdominoperineal resection or permanent colostomy - Any rectal cancer of the upper third (12–16 cm) requiring FU-CRT according to German S3 guideline recommendations (i.e. cT4, mrCRM+, extensive N + ) Patients with MRI-defined intermediate/high risk rectal cancer, but not eligible for TNT (oxaliplatin-containing) protocols: - any cT3 if the distal extent of the tumor is < 6 cm from the anocutaneous line, or - cT3c/d in the middle third of the rectum (≥6-12 cm) with MRI evidence of extramural tumor spread into the mesorectal fat of more than 5 mm (>cT3b), or - cT3 with clear cN1 based on strict MRI-criteria (see appendix) - cT4 tumors, or - T_any_ middle/low third of rectum with clear MRI criteria for N2 - mrCRM+ (≤1mm), or - Extramural venous invasion (EMVI + ) Trans-rectal endoscopic ultrasound (EUS) is additionally used when MRI is not definitive to exclude early cT1 disease in the lower third or middle third of the rectum. Spiral-CT of the abdomen and chest to exclude distant metastases. Aged at least 18 years. No upper age limit WHO/ECOG Performance Status ≤ 1 Adequate hematological, hepatic, renal and metabolic function parameters: - Leukocytes ≥ 3.000/mm^3^, ANC ≥ 1.500/mm^3^, platelets ≥ 100.000/mm^3^, Hb greater than 9 g/dl - Serum creatinine ≤ 1.5 × upper limit of normal - Bilirubin ≤ 2.0 mg/dl, SGOT-SGPT, and AP ≤ 3 × upper limit of normal
Exclusion Criteria	• Distant metastases (to be excluded by CT scan of the thorax and abdomen) Prior antineoplastic therapy for rectal cancer Prior radiotherapy of the pelvic region Major surgery within the last 4 weeks prior to inclusion Subject pregnant or breast feeding, or planning to become pregnant within 6 months after the end of treatment. Subject (male or female) is not willing to use highly effective methods of contraception during treatment and for 6 months after the end of treatment. On-treatment participation in a clinical study in the period 30 days prior to inclusion Previous or current drug abuse Other concomitant antineoplastic therapy Serious concurrent diseases, including neurologic or psychiatric disorders (incl. dementia and uncontrolled seizures), active, uncontrolled infections, active, disseminated coagulation disorder Clinically significant cardiovascular disease in (incl. myocardial infarction, unstable angina, symptomatic congestive heart failure, serious uncontrolled cardiac arrhythmia) ≤ 6 months before enrolment Prior or concurrent malignancy ≤ 3 years prior to enrolment in study (Exception: non-melanoma skin cancer or cervical carcinoma FIGO stage 0–1), if the patient is continuously disease-free Known allergic reactions on study medication Known dihydropyrimidine dehydrogenase deficiency Psychological, familial, sociological or geographical condition potentially hampering compliance with the study protocol and follow-up schedule (these conditions should be discussed with the patient before registration in the trial). History of severe hepatic impairment (e.g. Child-Pugh = Grade C) Moderate (Creatinine Clearance 30 to 49 mL/minute), severe (Creatinine Clearance < 30 mL/minute) renal impairment Neutropenia (neutrophil count < 1.5x10^9^/l) Known hypersensitivity to Anakinra or E. coli derived proteins, Anakinra or any of the components of the product Asthma Patients with clinically significant bacterial, fungal, parasitic or viral infection, which require acute therapy. Patients with acute bacterial infections requiring antibiotic use should delay screening/enrollment until the course of antibiotic therapy has been completed Patients with known active hepatitis B, C or who are HIV-positive or who are at risk for HBV reactivation. At risk for HBV reactivation is defined as hepatitis B surface antigen positive or anti-hepatitis B core antibody positive. Prior test results obtained as part of standard of care that confirm a subject is immune and not at risk for reactivation (i.e., hepatitis B surface antigen negative, surface antibody positive) may be used for purposes of eligibility and tests do not need to be repeated. Subjects with prior positive serology results must have negative polymerase chain reaction results. Subjects whose immune status is unknown or uncertain must have results confirming immune status before enrollment. Subjects who are already using the following medications will not be allowed: Tumor necrosis alpha inhibitors: Use on any of these biologics within 8 weeks of screening or baseline visit.IL -6 inhibitors: Use of any IL-6 inhibitors within 8 weeks of screening or baseline visit Janus Kinase inhibitors: Use of baricitinib, tofacinitib, upadacitinib, and ruxolitinib, oclacitinib, fedratinib, within 2 weeks from screening or baseline visit. Bruton's tyrosine kinase inhibitors: Ibrutinib, acalabrutinib, zanubrutinib CCR5 antagonist (CCR5 = C–C Chemokine Receptor Type 5; DMARD = Disease Modifying Anti-Rheumatic Drug): Leronlimab is also an immunomodulator. DMARDs: cyclosporine, cyclophosphamide, mycophenolic acid, chlorambucil, penicillamine, azathioprine: Use within 6 months prior to screening or baseline visit. Rituximab: Use of rituximab within 1 year of screening or baseline visit. Abatacept: Use of abatacept within 8 weeks of screening or baseline visit. Patients who have any severe and/or uncontrolled medical conditions or other conditions that could affect their participation such as severe impaired lung functions as defined as spirometry and DLCO that is 50% of the normal predicted value and/or O_2_ saturation that is 88% or less at rest on room air Patients under ongoing treatment with another investigational medication or having been treated with an investigational medication within 30 days (incl. live attenuated vaccine) of screening or 5 half-lives (whichever is longer) prior to the first dose of investigational product Patients receiving chronic, systemic treatment with corticosteroids or another immunosuppressive agent. Topical or inhaled corticosteroids are allowed History of any other disease, physical examination finding, or clinical laboratory finding giving reasonable suspicion of a disease or condition that contraindicates use of an investigational drug, or that might affect interpretation of the results of this study, or render the subject at high risk for treatment complications.

### Primary and Secondary objectives

The primary objective of this phase I drug-repurposing trial is the MTD of capecitabine in combination with the study medication anakinra (Kineret ®, 100 mg fixed dose, s.c., d-10 – d30) administered concomitantly with standard radiotherapy. To determine the MTD of capecitabine, a 3 + 3 dose-escalation design was chosen (Table 2). Maximum tolerated dose is defined as the highest dose of capecitabine at which 0 of 3, or no more than 1 of 6 evaluable patients experience pre-defined dose-limiting toxicity (DLT) per NCI CTCAE V5.0. At least 3 patients will be enrolled per dose level of capecitabine (500 mg/m^2^ bid, 650 mg/m^2^ bid and 825 mg/m^2^ bid, respectively). The criteria for dose escalation is that at least 3 patients completed treatment without DLT after 4 weeks. If DLT is observed, an additional 3 patients are included at the same dose level of capecitabine. The MTD is exceeded if DLT occurs in 2 of 6 patients (stopping dose). Once the MTD has been exceeded, another 3 patients at the previous dose level will be treated if there were only 3 patients treated at that dose level. The MTD is defined as the highest dose at which 6 patients were treated, of whom no more than one experienced a DLT. This dose will be recommended for further phase II efficacy testing. Dose escalation beyond 825 mg/m2 capecitabine is not intended. Accordingly, the number of patients included will be between 2 and 18.

**Table 2:** Characteristics of the 3 + 3 dose-escalation clinical trial design.

Cohort 1	Capecitabine 500 mg/m^2^, bid d1-30 during RT	• DLT 0/3 patients ≫ proceed to cohort 2 DLT 1/3 patients ≫ expand dose level to 6 patients DLT 1/6 patients ≫ proceed to cohort 2 DLT 2/6 patients ≫ MTD exceeded and trial terminated	
Cohort 2	Capecitabine 650 mg/m^2^, bid d1-30 during RT	• DLT 0/3 patients ≫ proceed to cohort 3 DLT 1/3 patients ≫ expands dose level to 6 patients DLT 1/6 patients ≫ proceed to cohort 3 DLT 2/6 patients ≫ MTD defined as 500 mg/m^2^, bid	
Cohort 3	Capecitabine 825 mg/m^2^, bid d1-30 during RT	• DLT 0/3 patients ≫ maximum dose reached (825 mg/m^2^, bid) DLT 1/3 patients ≫ expand dose level to 6 patients DLT 1/6 patients ≫ maximum dose reached (825 mg/m^2^, bid) DLT 2/6 patients ≫ MTD defined as 650 mg/m^2^, bid	

Secondary endpoints/clinical endpoints including disease-free survival (DFS), organ preservation (defined as survival with an intact rectum, no major surgery and no stoma) and quality of life/patient-reported outcomes. Detailed primary and secondary endpoints are given in Table 3. Disease-free survival is defined according to the international consensus recommendations on key outcome measures for organ preservation after (chemo)radiotherapy in patients with rectal cancer. Events are: no resection due to local progression/unfit patient, non-radical surgery of the primary tumor (R2 resection), locoregional recurrence after R0/1 resection of the primary tumor, non-salvageable local regrowth in case of W&W management (no operation or R2 salvage resection), metastatic disease before, at, or after surgery or W&W management, second primary colorectal cancer or other cancer, or death from any reason, whichever occurs first. This implies an optional W&W management for patients with cCR.

**Table 3:** Clinical endpoints of the ACO/ARO/AIO-21 trial.

Primary Endpoint	Analysis of safety and identification of the maximum tolerated dose (MTD) of capecitabine, administered concomitantly with standard radiotherapy in combination with Anakinra at a fixed dose of 100 mg s.c., will be the primary objective. A 3 + 3 design will be used. MTD is defined as the highest dose of capecitabine at which 0 of 3, or no more than 1 of 6 evaluable patients experience a dose-limiting toxicity (DLT) per NCI CTCAE V5.0. At least 3 patients will be enrolled per dose level of capecitabine (500 mg/m^2^ bid, 650 mg/m^2^ bid and 825 mg/m^2^ bid, respectively). The following will be considered DLT of capecitabine if they occur at any point whilst the patient is on study: Grade 4 neutropenia Grade 3 thrombocytopenia Grade 4 vomiting Grade 3 diarrhea lasting greater than 96 h despite adequate treatment and/or requiring CRT interruption of more than 5 days Grade 3 stomatitis Grade 3 hand-foot syndrome Grade 3 hepatic toxicity Grade 3 peripheral neuropathy
Secondary Endpoints	• Postoperative complications of (salvage) surgery Late toxicity assessment according to NCI CTCAE V.5.0 Rate of W&W with or without local regrowth Cumulative incidence of locoregional regrowth after cCR Rate of salvage surgery (LE/TME with or without APR/stoma) after locoregional regrowth Cumulative incidence of local recurrence after (salvage) surgery Cumulative incidence of distant recurrences Disease-free survival Overall survival Pathological TNM-staging R0 resection rate; negative circumferential resection rate Tumor regression grading according to Dworak Quality of TME according to MERCURY Quality of life and functional outcome based on treatment arm and surgical procedures/organ preservation Translational / biomarker studies

In addition, an extensive translational research program is implemented including blood, stool and tissue samples at multiple time points to further refine molecular prognostic and predictive profiling to ultimately identify subgroups for treatment stratification and/or escalation/de-escalation strategies.

### Treatment schedule

**Fig. 2:**
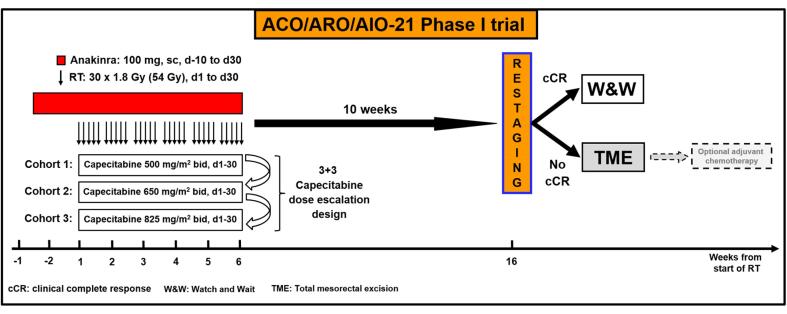
**Overview of the treatment schedule of the ACO/ARO/AIO-21 phase I clinical trial.** Patients with intermediate/high risk rectal cancer were treated with standard capecitabine-based CRT. The described RT dose is 54 Gy in 30 fractions (54/1.8 Gy). Anakinra is initiated on d-10 and administered daily (100 mg fixed dose, s.c.) until the last fraction of RT (d30). Capecitabine is given concomitantly on d1-30 at predefined dose levels, bid. Response assessment (RA) is scheduled 10 weeks after treatment. Primary NOM for patients with cCR includes an intensified local follow-up regime. In case of non-cCR, immediate TME surgery will be performed followed by optional adjuvant chemotherapy.

Patients are subjected to standard preoperative CRT with oral capecitabine during RT. Intensity modulated radiotherapy (IMRT) or volumetric modulated arc therapy (VMAT) is applied as follows: 25 × 1.8 Gy (total 45 Gy) to the primary tumor, the mesorectum and the internal iliac lymph nodes up to the interspace of S2-3 in low rectal tumors without suspected lymph node involvement, or the interspace or L5-S1, respectively. The inferior border is at least 3 cm below the primary tumor. A sequential boost of 5 × 1.8 Gy (total 9 Gy) is applied to gross tumor volume (GTV) plus margins. Capecitabine is given from d1-30 according the aforementioned 3 + 3 dose-escalation design. Anakinra (100 mg fixed dose, s.c.) is administered from d-10 (10 days prior to RT) to the last day of RT (d30). Response assessment (RA) including digital rectal examination (DRE), MRI of the pelvis and endoscopy is scheduled 10 weeks after CRT. Patients with cCR are assigned to primary NOM and W&W option with close follow-up and restaging examinations. In case of non-CR, immediate TME surgery will be recommended followed by an optional adjuvant chemotherapy depending on the postoperative pathological risk factors Fig. 2.

### Safety analyses

Continuous evaluation of safety data through close monitoring will be performed. Adverse events will be summarized by treatment, body system and preferred term, intensity, and causal relationship to study agent and their frequencies and percentages will be reported. The safety report is according to the International Council for Harmonisation of Technical Requirements for Registration of Pharmaceuticals for Human Use (ICH) E3 “Structure and content of Clinical Study Reports” (CPMP/ICH/137/95). Events will be recorded from the time that the subject has received the first dose of study treatment until the End-of-Treatment visit.

### Response Assessment, efficacy evaluation and Follow-up procedures

Non-pathological response assessment is scheduled 10 weeks after the completion of therapy and includes DRE, MRI of the pelvis, rectoscopy (optional biopsy) and CEA monitoring. Complete and near complete clinical response is defined as described in Table 4.

**Table 4:** Definition of cCR, near cCR and poor response 10 weeks after CRT.

	cCR	Near cCR	Poor Response
DRE	No palpable tumor	Small and smooth mucosal irregularities	Palpable tumor mass
Rectoscopy	Flat, white scar with or without telangiectasia. No ulcer. No nodules.	Residual ulcer or small mucosal nodules or minor mucosal abnormalities. Mild persisting erythema of the scar.	Visible macroscopic tumor
MRI	No residual suspicious lymph nodes	Regression of lymph nodes with no malignant enhancement features but size greater than 5 mm.	No regression of suspicious lymph nodes.

Follow-up schedule for primary NOM or surgical management after CRT is detailed in Table 5 and includes clinical and radiological response assessment as well as clinical and laboratory examinations. After surgery, ypTNM, resection status, circumferential resection margins (CRM), tumor regression grading (TRG) according to Dworak, and TME quality according to MERCURY are reported.

**Table 5:** **Follow-up schedule** Time points are marked with an x. Only after surgery x* and NOM (x), respectively.

**Evaluation**	**Time after Completion of Therapy**												
	**Weeks**	**Months**											
	**10**	**3**	**6**	**9**	**12**	**15**	**18**	**21**	**24**	**30**	**36**	**48**	**60**
Physical Examination	x	x	x	x	x	x	x	x	x	x	x	x	x
Serum CEA	x	x	x	x	x	x	x	x	x	x	x	x	x
Rectoscopy	x	(x)	x	x	x	x	x	x	x	(x)	(x)	(x)	(x)
Colonoscopy			x										x
Pelvic MRI	x	(x)	(x)	(x)	(x)		(x)		(x)		(x)	(x)	(x)
CT Scan/Chest X-Ray		x*			x				x		x	x	x
Abdomen Sonography		x*	x	x*	x	x*	x	x*	x	x	x	x	x
AE/SAE Assessment	x	x	x	x	x	x	x	x	x	x	x	x	X
EORTC QLQ-C30, -CR29, -CPIN20, Wexner Score	x	x	x		x		x		x		x		x

### Statistical aspects

Primary objective of this phase I study is to define the MTD of capecitabine administered concomitantly to RT in combination with fixed doses of the IL-1RA anakinra. The sample size is driven by the primary objective of the study based on a 3 + 3 design and ranges between two and 18 patients [12]. Recruitment is planned over 2 years and all patients will be followed up for at least 3 years.

### Time schedule

Preparation began in Q4 2020. The first patient was enrolled in 08/2021. Final report will be expected Q2 2026.

## Discussion

The ACO/ARO/AIO-21 phase I drug re-purposing trial will evaluate the safety and tolerability of combining the IL1-RA anakinra with preoperative fluoropyrimidine-based CRT in patients with rectal cancer. Potential benefits for patients with rectal cancer participating in the present drug-repurposing trial include access to the study medication anakinra, which (a) is not available outside of a clinical trial for rectal cancer but is approved for standard use in rheumatoid arthritis and other nonmalignant diseases, where (b) it has been used for more than 15 years in approximately 150,000 patients worldwide with proven safety, (c) has shown clinical efficacy in combination with chemotherapy for malignancies, including metastatic colorectal cancer, (d) based on preclinical studies, may increase the efficacy of fluoropyrimidine-based CRT, resulting in enhanced tumor response and local control, but may also reduce distant metastases to improve long-term oncologic outcomes.

The evolution of neoadjuvant treatment concepts has gradually led to significant improvements in local control, DFS, and complete response rates [13]. Based on the results of the RADIPO, UNICANCER-PRODIGE23 and CAO/ARO/AIO-12 clinical trials, emerging evidence supports TNT concepts in treating high-risk rectal cancer, incorporating upfront combinations of radiation and additional chemotherapy. Compared with standard fluoropyrimidine-based CRT, TNT concepts have significantly improved DFS rates up to 78% and increased pathological complete response (pCR) rates between 25% and 28% [4], [5], [6]. Nevertheless, not all patients are eligible for TNT with intensive chemotherapy regimens, and a substantial subgroup of patients exhibits therapy resistance to RT/CRT resulting in disease progression and impaired survival, indicating the need for extensive translational research and new therapy approaches [14], [15], [16], [17].

Molecular profiling of rectal cancer has not yet been routinely established for accurate prediction of treatment response and outcomes, and mechanisms that drive therapy resistance are poorly understood [18], [19], [20]. Recently, components of TME have become increasingly recognized as important modulators of disease progression and treatment response [21]. Cancer-associated fibroblasts, with their contributory role in cancer progression and therapy resistance, are a key component within the TME. Our group could recently demonstrate the critical role of CAFs in mediating therapy resistance upon tumor-derived IL-1α signaling in a murine rectal cancer model or PDOs. After inflammatory polarization of CAFs (iCAFs), irradiation resulted in CAF senescence and ECM accumulation [11]. The accumulation of ECM molecules creates a hypoxic environment, which in turn promotes tumor angiogenesis and therapy resistance. The increase in ECM molecules further causes matrix stiffening, which forms a physical barrier deteriorating drug delivery and facilitating immune evasion by impeding T cells [22]. Moreover, certain subsets of CAFs are capable to influence immune infiltration and to promote an immunosuppressive and pro-tumorigenic TME via a specific immunomodulatory secretome [23], [24]. Dysregulation of IL-1 has been observed in almost all types of human malignancies, implicating its important contribution to tumorigenesis and cancer progression [25], [26]. In our murine rectal cancer model, IL-1 inhibition using the IL-1 receptor antagonist (IL-1RA) anakinra has significantly sensitized tumors to RT and abrogated distant metastases [11]. We hypothesize that starting anakinra 10 days before CRT could reconstitute the TME by inhibiting intrinsic inflammatory CAF polarization and thus facilitating an improved response to therapy.

The safety profile of anakinra has been studied for years, as it is approved for the treatment of autoimmune and inflammatory diseases such as rheumatoid arthritis, juvenile and adult Still's disease, and cryopyrin-associated periodic syndrome (CAPS) [27], [28], [29]. Most common adverse events are injection site reactions, cephalgia, neutropenia and thrombocytopenia. Opportunistic infections in patients treated with Anakinra are rare, including in populations at high risk for reactivation of M. tuberculosis infections. During controlled trials, more viral upper respiratory tract infections have been reported in patients treated with anakinra compared to patients treated with placebo. There are two spurious reports of anakinra-related hepatotoxicity in patients with Still’s disease; however, withdrawal of Anakinra restored normal liver function [30], [31]. The safety, tolerability and efficacy of the combination of fluorouracil (5FU) and the monoclonal VEGF-A antibody bevacizumab plus anakinra was already evaluated in a single-arm phase II study in patients with refractory metastatic colorectal cancer (IRAFU). In the trial by Isambert et al., 32 patients were enrolled. The addition anakinra resulted in a median progression-free survival (PFS) of 5.4 months (95% CI, 3.6–6.6) and overall survival (OS) of 14.5 months (95% CI, 9–20.6) without increasing toxicity rates. Response according the Choi criteria was observed in five patients, while 22 patients had stable disease. Most common grade 3 adverse events were neutropenia in eight patients (25%) and digestive side effects in seven (21.9%) patients [32]. Overall, this study demonstrated a manageable safety profile and promising response rates in a heavily pretreated patient cohort. Meanwhile, final results of further clinical trials testing anakinra in various malignancies are pending (advanced/recurrent/metastatic malignancies (NCT01624766), metastatic tumors (NCT00072111), metastatic breast cancer (NCT01802970), pancreatic cancer (NCT02550327, NCT02021422)). Preliminary results suggest a manageable profile of adverse events for anakinra in combination with cytotoxic therapies [33], [34], [35].

In summary, the above findings provide a strong clinical and pre-clinical rationale for combining standard fluoropyrimidine-based preoperative CRT with the IL-1RA anakinra targeting the TME in a primary setting, which could significantly improve tumor response and clinical outcomes. The ACO/ARO/AIO-21 phase I drug re-purposing trial will therefore assess the safety and tolerability of the IL-1RA anakinra in combination with fluoropyrimidine-based CRT in patients with LARC, with the view of expanding into a randomized phase II trial.

## Trial Registration

EudraCT-No.: 2021-000562-15.

ClinicalTrials.gov Identifier: NCT04942626; first posted on June 28, 2021.

## Funding

The trial is sponsored by the LOEWE-Zentrum Frankfurt Cancer Institute (FCI) [III L 5 - 519/03/03.001 – (0015), PI: Emmanouil Fokas].

## Declaration of Competing Interest

The trial is sponsored by the LOEWE-Zentrum Frankfurt Cancer Institute (FCI) [III L 5 - 519/03/03.001 – (0015), PI: Emmanouil Fokas]. The authors have no further conflicts of interest to declare.
